# Variability in the chemistry of private drinking water supplies and the impact of domestic treatment systems on water quality

**DOI:** 10.1007/s10653-016-9798-0

**Published:** 2016-01-25

**Authors:** E. L. Ander, M. J. Watts, P. L. Smedley, E. M. Hamilton, R. Close, H. Crabbe, T. Fletcher, A. Rimell, M. Studden, G. Leonardi

**Affiliations:** 1 Inorganic Geochemistry, Centre for Environmental Geochemistry, British Geological Survey, Keyworth, Nottingham NG12 5GG UK; 2 Groundwater Science, British Geological Survey, Keyworth, Nottingham NG12 5GG UK; 3 Environmental Change Department, Centre for Radiation, Chemical and Environmental Hazards (CRCE), Public Health England, Chilton, Didcot, Oxfordshire OX11 0RQ UK

**Keywords:** Arsenic, Manganese, Point-of-entry treatment, Water quality standards, Public health

## Abstract

Tap water from 497 properties using private water supplies, in an area of metalliferous and arsenic mineralisation (Cornwall, UK), was measured to assess the extent of compliance with chemical drinking water quality standards, and how this is influenced by householder water treatment decisions. The proportion of analyses exceeding water quality standards were high, with 65 % of tap water samples exceeding one or more chemical standards. The highest exceedances for health-based standards were nitrate (11 %) and arsenic (5 %). Arsenic had a maximum observed concentration of 440 µg/L. Exceedances were also high for pH (47 %), manganese (12 %) and aluminium (7 %), for which standards are set primarily on aesthetic grounds. However, the highest observed concentrations of manganese and aluminium also exceeded relevant health-based guidelines. Significant reductions in concentrations of aluminium, cadmium, copper, lead and/or nickel were found in tap waters where households were successfully treating low-pH groundwaters, and similar adventitious results were found for arsenic and nickel where treatment was installed for iron and/or manganese removal, and successful treatment specifically to decrease tap water arsenic concentrations was observed at two properties where it was installed. However, 31 % of samples where pH treatment was reported had pH < 6.5 (the minimum value in the drinking water regulations), suggesting widespread problems with system maintenance. Other examples of ineffectual treatment are seen in failed responses post-treatment, including for nitrate. This demonstrates that even where the tap waters are considered to be treated, they may still fail one or more drinking water quality standards. We find that the degree of drinking water standard exceedances warrant further work to understand environmental controls and the location of high concentrations. We also found that residents were more willing to accept drinking water with high metal (iron and manganese) concentrations than international guidelines assume. These findings point to the need for regulators to reinforce the guidance on drinking water quality standards to private water supply users, and the benefits to long-term health of complying with these, even in areas where treated mains water is widely available.

**Electronic supplementary material:**

The online version of this article (doi:10.1007/s10653-016-9798-0) contains supplementary material, which is available to authorized users.

## Introduction

Drinking water can be a major dietary source of some beneficial trace elements, but can also be a significant source of exposure to substances which are harmful to health in excess quantities, such as arsenic (As), fluoride (F) and nitrate (NO_3_) (WHO 2011a). The presence of elements such as iron (Fe) and manganese (Mn) can cause both aesthetic problems and, at higher concentrations, can have potentially harmful health effects (WHO 2011a). The WHO guideline values (GV) form the basis of many national and international water quality standards, including European Union legislation (Directive 98/83/EC, Council of the European Union 1998). The GVs are periodically reviewed and revised, which generates considerable debate on the merits of those revisions (e.g., Frisbie et al. 2012; Ljung and Vahter 2007; Vinceti et al. 2013).

The chemical quality of drinking water sourced from groundwater is known to vary spatially as a result of: variations in aquifer geology and chemistry; treatment works method(s); and reaction between the water and distribution and/or plumbing systems. Bottled waters similarly vary, predominantly from natural processes, but may also be influenced by leaching of the bottle itself (Smedley 2010; Reimann and Birke 2010).

Spatial differences in groundwater chemistry vary over a scale determined by factors including the aquifer extent and heterogeneity, groundwater flow paths and residence times, and the intensity of external inputs, such as anthropogenic contaminants (Shand et al. 2007; Smedley 2010; DeSimone et al. 2009). The occurrence of high concentrations of trace elements, such as As, can be very unpredictable at the local scale, or subject to temporal variation (Ayotte et al. 2015). This makes the acquisition of sample data essential to understand chemical water quality in a given area and enable lifetime exposure risks to be quantified (Hough et al. 2010; Nuckols et al. 2011). Globally, in areas where groundwater used as drinking water has very high As concentrations, it is recognised as a cause of high morbidity and premature mortality rates (Murcott 2012; Smith et al. 2000). Even at lower drinking water As concentrations (<100 µg/L), there is growing evidence of associations with population-level health outcomes (Leonardi et al. 2012; Gilbert-Diamond et al. 2013). Concentrations of other analytes, such as Mn, NO_3_ and uranium (U), in drinking water have been studied to ascertain whether they are associated with specific public health outcomes (e.g., Frisbie et al. 2009; Ljung and Vahter 2007; Fewtrell 2004). It is recognised that mixtures can increase overall toxicity (DeSimone et al. 2009), but evidence of effects is not widely reported in the literature.

Where analytes exceed quality guidelines in private drinking water supplies, treatment systems appropriate to domestic dwellings can be used to decrease the concentrations to acceptable levels (Slotnick et al. 2006; Möller et al. 2009). Indeed specially designed guidance may be available on a local level to encourage water quality testing, with installation of appropriate treatment (e.g., Scottish Executive 2006; Charrois 2010). It is emphasised both in research outputs and public communications that there is a need for domestic treatment systems to be maintained appropriately, to ensure efficacy (Möller et al. 2009; Scottish Executive 2006; Flanagan et al. 2015). Treatment systems can be point-of-entry, or point-of-use (e.g., under kitchen sink), and either may have benefits. However, it has been shown that greater reduction of householder exposure to As is achieved by having point-of-entry treatment rather than just at the primary cooking and drinking tap (Spayd et al. 2015), and point-of-entry intervention would be required to prevent corrosion of copper (Cu) plumbing systems by low-pH water. Point-of-use systems have the potential cost saving of treating much smaller volumes of water and may be more suitable for some parameters, such as NO_3_ removal (Scottish Executive 2006).

Within the UK, drinking water quality standards are the national implementation of EC Directive 98/83/EC, and some national standards (DEFRA 2009). Public water utilities, and those distributing water from private water supplies, are closely monitored for compliance with the national regulations (Drinking Water Inspectorate 2015). These employ treatments which will alter the concentration of many elements (Dinelli et al. 2012). However, at single domestic dwellings with private water supplies, the householder is not compelled to undertake water quality testing or improvement, or to inform the authorities of these actions and their outcomes (Drinking Water Inspectorate 2015). This is also the case elsewhere in Europe and in North America (e.g., Zheng and Ayotte 2015; Charrois 2010). There is, therefore, a dearth of information on private water supply quality from the perspective of inorganic chemistry, especially from surveys with statistically robust sampling design.

Private water supplies have the potential to be important from a public health perspective, due to the potential for poor quality water and because there are an estimated 37,717 supplies, predominantly in rural areas of England (Drinking Water Inspectorate 2015). Whilst a recent publication has compiled data reported by the regulators (local authorities) in England (Drinking Water Inspectorate 2015), the data for the two smallest types of supply (‘Regulation 10’ and ‘Single Domestic Dwellings’) are still characterised by low sample numbers (e.g., only 249 As analyses at single domestic dwellings throughout England in a 5-year period) even though these categories of supply are generally the most common. Older summaries of regulator held data do not include parameters such as As (Harrison et al. 2000). Systematic bias may be implicit in the national compilation data for these smaller supply categories, particularly if they disproportionately represent households who have sought help from the local authority because they suspect a problem. Such bias was observed in a comparison of local authority and random design groundwater surveillance As data in New Hampshire, USA (Peters et al. 1999).

There are many studies of the microbial quality in private water supplies in Britain, showing high exceedances (≥50 %) (Fewtrell et al. 1998; Reid et al. 2003; Galbraith et al. 1987; Shepherd and Wyn-Jones 1997; Richardson et al. 2009; Rutter et al. 2000; Said et al. 2003; Risebro et al. 2012). However, there are few studies which assess chemical quality parameters at the point of consumption (rather than abstraction). Those that do are either focused on NO_3_ (Reid et al. 2003; Chilvers et al. 1984), and individual high concentration incidents reported to the authorities, or public water supply chemical incidents (Paranthaman and Harrison 2010). Groundwater in England has been shown to be highly variable for a range of parameters covered by drinking water quality standards (Shand et al. 2007), including one small study in part of Cornwall (Smedley and Allen 2004). Tracts of England, including Cornwall in the south-west, are characterised by typically high concentrations of As, and other elements in the surface environment (soils, stream sediments, made ground). These are naturally occurring, and a legacy of mining and smelting activities from the eighteenth and nineteenth centuries, with As one of the economically important commodities (Ander et al. 2013; Aston et al. 1975; Abrahams and Thornton 1994). Given the well-documented concerns over As in drinking water, this represents a knowledge gap in an area of otherwise widespread high environmental As concentrations.

In order to address this data deficiency, a representative survey was implemented to collect private drinking water supply samples across Cornwall, an area of 3500 km^2^. Cornwall was selected due to the combination of awareness of high concentrations of As and other elements in the surface environment, and a large number of private water supplies, with the 2014 estimate at 3811 (Drinking Water Inspectorate 2015). The primary aim of the work presented here is to provide an understanding on the chemical quality of private drinking water supplies, and how these relate to water quality guideline values. The secondary aim is to assess the treatment systems being used, and any impact these are having on the chemical quality of the drinking water. The overarching objective is to help quantify human exposure to chemicals in private drinking water supplies in the UK and identify any potential public health risks, as part of Public Health England’s (PHE) Environmental Public Health Tracking (EPHT) programme. A risk assessment based on geology, and population exposure estimation, is also being conducted as part of a series of studies examining the public health risk of chemicals in private water supplies.

## Materials and methods

### Project design and communications

**Fig. 1:**
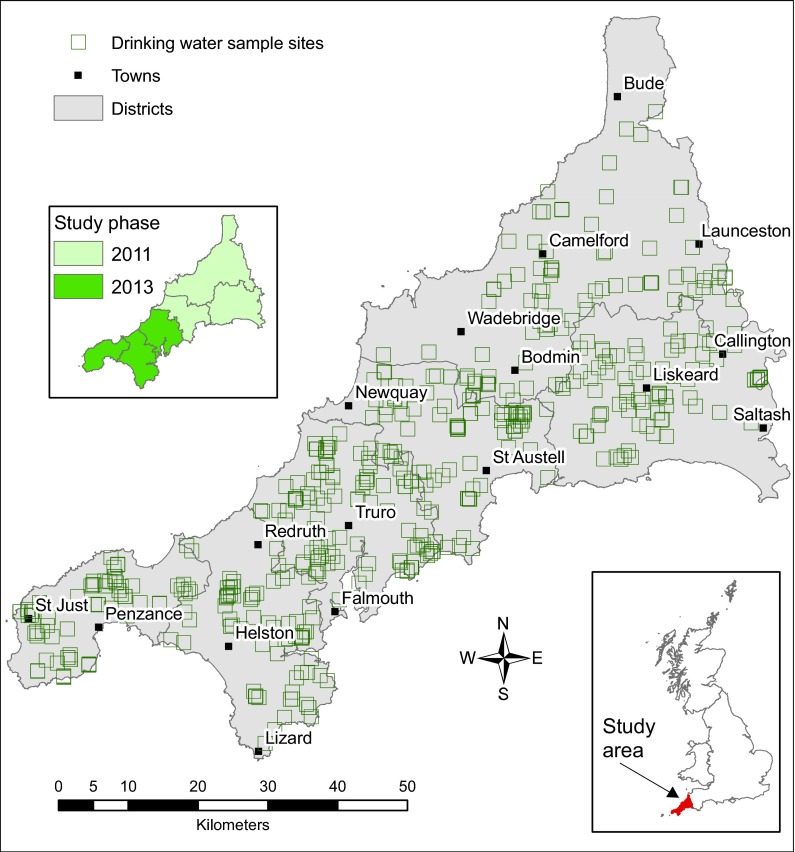
Location map of Cornwall, with the study sites and sampling phases

Sampling of private drinking water supplies across Cornwall was undertaken in two phases, spring 2011 and spring 2013 (Fig. 1), with approximately equal numbers of samples collected in each campaign. Householders identified in local authority (Cornwall Council) records as using private water supplies were invited to participate. Households were randomised from the original list. Samples were collected at those properties where householders could be contacted and then volunteered to participate. Additional properties were identified by word-of-mouth recommendation when field teams were operating, since not all private water supply abstraction points are registered on public authority databases. The volunteer recruitment and appointment booking system was operated by PHE (formerly Health Protection Agency, HPA). Attempts were made to arrange appointments when householders were present. Whilst successful in the majority of cases, this was not always possible and then the most suitable (as guided by the householder) available sampling point was taken, such as from a tap on an outer wall of the house.

Sampling design, sampling, data analysis and data reporting were undertaken by the British Geological Survey (BGS). Individual data feedback to participants was provided through a letter containing specific guidance which was developed by PHE along with BGS and Cornwall Council. The feedback letter was sent from the Local Authority, as the regulator for private water supplies in England. Participants were given advice on any potential health risks and suggested corrective actions where they had one or more exceedances of the water quality standards, and all participants were provided with appropriate contact details for any follow-up enquiries. This study design and analysis specifically did not include the analysis of any organic, microbial or radiological properties of the water samples, nor an exhaustive suite of inorganic constituents covered by the regulations (DEFRA 2009). This study design specifically included only supplies using groundwater.

### Sample collection and data recording methods

Water sample collection methods followed standard protocols used at BGS for groundwater and tap water sampling (e.g., Shand et al. 2007; Smedley et al. 2014b), as summarised in Table 1. Groundwater samples were collected near to the wellhead and filtered on collection to 0.45 µm, in order to capture information about the dissolved component. Tap waters (synonymous with ‘drinking water’ samples in this paper) were not filtered at collection, since this would not be representative of water drawn for drinking or cooking; thus, they may include fine particulates. There were 29 taps at which paired filtered and unfiltered samples were both collected. Data from this filtering test permit direct comparison of the effect of filtering and possible particulate transport in drinking water samples.

**Table 1:** Sample collection and analytical method summary

Method	Drinking water	Groundwater
Sample point	Tap used as primary drinking and cooking water supply. Where householder absent, most accessible tap representing water used by household. Tap drawn for 3 min at a moderate steady flow before collection/measurement	First available tap point for boreholes: prior to storage, treatment or air ingress. Householder questioned on frequency of use—any rarely used sources run for longer than those used several times a day. Direct analysis and sample retrieval from dug wells
Unstable parameters measured	Temperature, dissolved oxygen, specific electrical conductance (SEC), pH and redox potential (Eh) using a Hanna Instruments 9828 multimeter into a small beaker, thoroughly rinsed before measurements. Alkalinity was measured by titration at site using a Hach titrator, taking using an average of at least two measurements	Temperature, dissolved oxygen, specific electrical conductance (SEC), pH and redox potential (Eh) using a flow-through cell (from pumped boreholes) and Hanna Instruments 9828 multimeter. Alkalinity was measured by titration at site, using a Hach titrator, taking using an average of at least two measurements
Sample for analysis	2 × LDPE 30 mL Nalgene^®^ bottle filled with unfiltered sample. Bottle rinsed with ~5 mL of sample, discarded then filled	2 × LDPE 30 mL Nalgene^®^ after passing through a 0.45 µm Acrodisc^®^ syringe filter (pre-wrapped) and 20 mL Plastipak syringe. Syringe rinsed with ~5 mL sample, then sample bottle rinsed with ~5 mL filtered sample, discarded and then filled
Sample preservation	1 % v/v HNO_3_ (at the end of the day) + 0.5 % v/v HCl (on receipt in the laboratory) for the acidified sample, and refrigerated. Refrigeration for the unacidified sample	
Unacidified sample analysis	Ion chromatography for 7 anions (Cl^−^, SO_4_^2−^, NO_3_^−^, Br^−^, F^−^, HPO_4_^2−^ and NO_2_^−^)	
Acidified sample analysis	ICP-MS for 57 elements (Li, Be, B, Na, Mg, Al, Si, P, S, K, Ca, Ti, V, Cr, Mn, Fe, Co, Ni, Cu, Zn, Ga, As, Se, Rb, Sr, Y, Zr, Nb, Mo, Ag, Cd, Sn, Sb, Cs, Ba, La, Ce, Pr, Nd, Sm, Eu, Tb, Gd, Dy, Ho, Er, Tm, Yb, Lu, Hf, Ta, W, Tl, Pb, Bi, Th, U)	
Additional information acquired by questionnaire recorded	Sample location, date and time; householder reported point-of-entry/use treatment and storage or pressurised system; location and nature of the supply headworks; relative volumes/frequency of abstraction and water use (e.g., part-time occupancy/normal domestic household/household plus livestock watering)	

Contextual data were recorded at site by interview with the householder (Table 1). Treatment system information was also recorded, with knowledge of systems varying greatly between households. Some participants had no knowledge of any treatment occurrence, or its purpose, whilst others were undertaking regular maintenance of the systems themselves. Where water was provided from off-property, the nature of the treatment installation could not always be observed by the sampler.

### Analytical methods

Unstable parameters were measured using a multi-probe (pH, temperature, conductivity, dissolved oxygen, redox potential) and by titration (alkalinity) at site (Table 1). The multi-probes were calibrated and checked each day. The collected samples were kept in cool conditions in the field and refrigerated (4 °C) from the evening of collection onwards. Chemical analyses were undertaken in the ISO 17025:2005 accredited BGS Inorganic Geochemistry laboratories by the analytical methods summarised below and in Table 1. More detail on the standard methods and quality control used are provided in O’Reilly et al. (2010).

Acidification with 1 % v/v HNO_3_ was undertaken on the ‘acidified’ aliquot on the evening of collection, and further acidification with 0.5 % v/v HCl was undertaken on those samples, on their return to the BGS laboratory. Analysis of the acidified samples, for 57 elements (Table 1), used an Agilent 7500cx series inductively coupled plasma mass spectrometer (ICP-MS). The ICP-MS was optimised before each analytical run using a 5 µg/L tuning solution consisting of lithium, cerium, yttrium and thallium (SPEX CertiPrep, USA). A mixed internal standard solution containing scandium, germanium, rhodium, indium, tellurium and iridium was added to the samples at a fixed ratio of approximately 1:10 via a T-piece. A Certified Reference Material (CRM, NIST 1643e) was run 33 times interspersed through the analytical programme, with all certified elements having accuracies of 95–105 % of the certified value.

Anion analysis (Table 1) of unacidified samples was by ion chromatography (IC) using a Dionex DX-600 Ion Chromatograph (Thermo Fisher), with an AG14 guard column and an AS14 analytical column, with an injection volume of 100 μL. All analytes have an uncertainty <10 %.

Field blank waters (filtered and unfiltered), as well as ‘blind’ field duplicate and CRM (SLRS-5) samples, were used to monitor analytical performance in addition to, and independent of, the laboratory quality control assessments at 10 % of samples. These further supported the use of these data as blank concentrations did not indicate contamination problems, and duplicate analyses demonstrated that variance was dominated by between site sources (≥94 %), not analytical or sampling sources.

### Data storage and presentation

Field data and analytical records are stored in a securely held relational Access database (Microsoft Access 2010, Microsoft Corporation, Redmond, WA, USA). Statistical analysis and graphing used R core programme (R Core Team 2015) and StatDA package (Filzmoser 2015), via RStudio version 0.98.1103 (RStudio, Boston, USA), and in SigmaPlot version 11.0 (Systat software, San Jose, CA, USA). Mapping was undertaken in ArcGIS version 9.3 (ESRI, Redlands, CA, USA).

The chemical parameters reported here are primarily those for which there is a water quality standard, referred to as the Prescribed Concentration/Value or PCV (DEFRA 2009):

- ‘Chemical parameters’—antimony (Sb), As, boron (B), cadmium (Cd), chromium (Cr), Cu, F, lead (Pb), nickel (Ni), NO_3_, nitrite (NO_2_) and selenium (Se);
- ‘National requirement’—aluminium (Al), Fe, Mn, sodium (Na); and,
- ‘Indicator parameters’—chloride (Cl), conductivity, pH, and sulphate (SO_4_).

Additionally, WHO has guideline values (GV) for barium (Ba), beryllium (Be), bromide (Br), molybdenum (Mo) and U, which it is useful to consider, from the perspective of better understanding the ramifications should they be introduced in a revision of national, or EU, guidelines, e.g., as by Smedley et al. (2014b).

### Drinking water samples

Samples were collected from 515 distinct sources, and individual analytical results have been provided to all participants. From these, 497 samples were classified as drinking water for this study, as the sample was used for drinking and/or cooking water. The majority of properties sampled were single domestic dwellings, consistent with the general reported trend. Groundwater samples were also collected where possible. Groundwater data are only presented in this paper where they form part of a groundwater and drinking water sampling pair at a property, either providing information on before and after treatment composition (*n* = 138), or untreated sample pairs (*n* = 24). Sample data are not used here where water type could not be confirmed.

Boreholes were the most common method of extracting water, at 82 % (*n* = 406), with traditional large-diameter wells accounting for 12 % (*n* = 62) and spring capture 3 % (*n* = 14). There were 15 properties (3 %) where the source could not be confirmed. The depth and age of borehole and well supplies was not always known, but where this information could be returned, it ranged from many centuries to within the last decade (particularly where dwellings have been renovated). Wells were generally shallow (minimum <2 m below surface), whilst boreholes were reported up to ~100 m deep. During the survey, disused well covers were observed at many properties using boreholes. Anecdotally, it was reported that boreholes had been installed to replace shallow wells, with the most commonly cited reasons for this being to reduce risk from microbial ingress, or to ensure security of yield during dry summer months when some shallow wells were liable to dry out.

### Treatment systems used

**Fig. 2:**
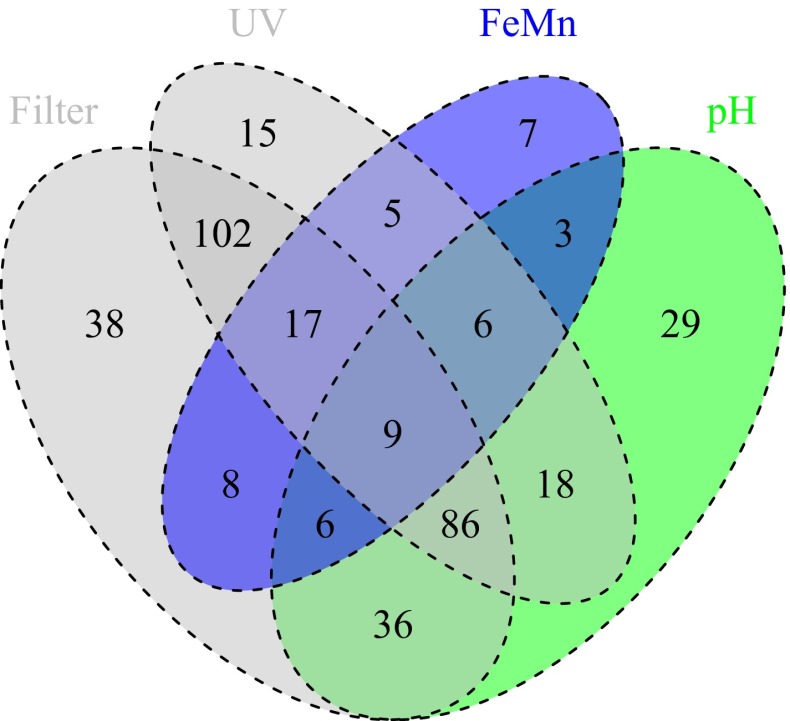
Venn diagram of treatment options reported by the study households. The four most common treatment options are shown

Treatment could be recorded for 487 of the 497 (98 %) drinking water samples. Of these, 21 % (*n* = 102) were untreated, and 47 % (*n* = 229) had no disinfection system in place using UV or, rarely, chlorination (*n* = 5). This is a higher proportion than reported previously in a data compilation (Rutter et al. 2000). Physical filtration (5 µm) was recorded for 302 (62 %) samples. One or more chemical treatments were reported for 47 % (*n* = 230) of samples, and these were dominated by pH adjustment (*n* = 193) and Fe/Mn removal (*n* = 61). Of the four most frequently employed systems, the multiple combinations (Fig. 2) show filtration and UV in combination to be the most frequent permutations, followed by those treatments in combination with pH adjustment.

It is recognised that self-reporting of treatment systems may give rise to a proportion of samples where the householder wrongly identifies the system in place, or maintenance recommendations have not been followed. For this reason, data conforming to the following criteria have been used to assess the effects of pH adjustment on wider chemical properties: (1) starting pH < 6.5 and finished pH > 8.5 (*n* = 9), as a pH adjusted dataset; or (2) both pH values <6.0 (*n* = 14), with no record of Fe/Mn removal treatment (which may inherently include pH adjustment), as a ‘control’ dataset with no treatment. The starting pH value of these groups were not significantly different (two-sample *t* test, *p* = 0.44) and neither subset was skewed (skew = 0.1 and 0.8 for low and high drinking water pH, respectively). Whilst these sample data do not arise from a controlled experiment with all other variables being held equal, they can be used as indicators of typical changes taking place.

## Results

**Fig. 3:**
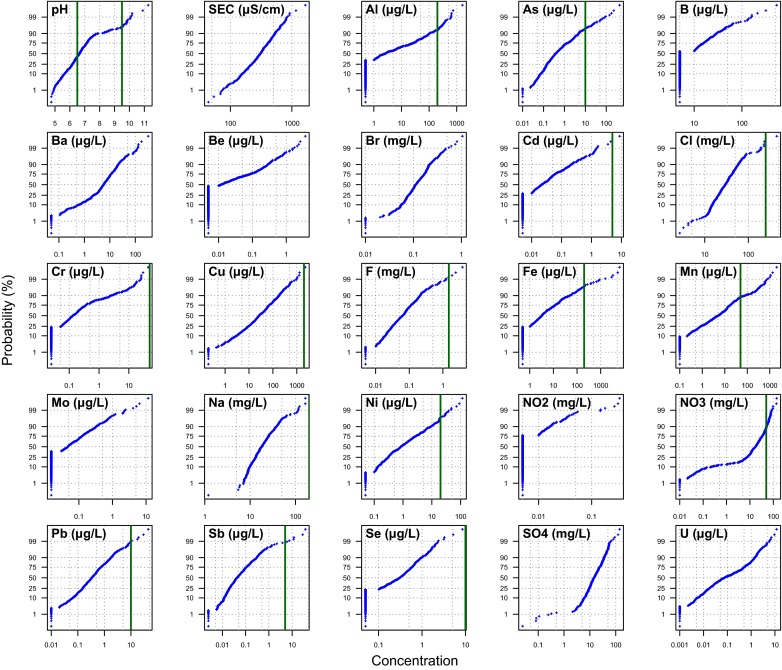
Cumulative probability plots of drinking water data. Concentration axis is on a log_10_-transformed scale, other than for pH. *Vertical green line*-PCV or WHO values, where these are below the axis maximum value

Table 2 and Fig. 3 data summaries show that for all 25 parameters reported here the analytical techniques used have sufficient sensitivity to measure the majority of tap water concentrations, and in all cases have a detection limit at least 100-fold lower than the threshold value of interest. Concentrations generally have a total range across three or four orders of magnitude in total. Some parameters exhibit strongly bimodal data distributions, particularly pH and NO_3_ (Fig. 3).

**Table 2:** Statistical summary of the drinking water sample chemical data which have a PCV or WHO value

	pH^a^	SEC^b^	Al	As	B	Ba	Be	Br	Cd	Cl	Cr	Cu	F
Method	Metre	Metre	ICP-MS	ICP-MS	ICP-MS	ICP-MS	ICP-MS	IC	ICP-MS	IC	ICP-MS	ICP-MS	IC
Units	–	µS/cm	µg/L	µg/L	µg/L	µg/L	µg/L	mg/L	µg/L	mg/L	µg/L	µg/L	mg/L
Detection limit			1	0.02	10	0.1	0.01	0.02	0.01	0.05	0.05	0.4	0.01
Minimum	4.75	44	<1	<0.02	<10	<0.1	<0.01	<0.02	<0.01	2.67	<0.05	<0.4	<0.01
5th percentile	5.38	131	<1	0.05	<10	0.19	<0.01	0.04	<0.01	13.0	<0.05	1.36	0.01
25th percentile	6.17	223	<1	0.15	<10	2.31	<0.01	0.08	0.01	20.6	0.05	9.47	0.03
50th percentile	6.64	306	2.65	0.38	<10	5.69	0.01	0.11	0.02	29.6	0.13	26.5	0.06
75th percentile	7.14	410	26.5	1.43	15.9	11.0	0.09	0.17	0.06	42.2	0.37	69.7	0.11
95th percentile	9.39	638	230	11.0	44.7	35.0	0.51	0.28	0.46	71.5	8.85	274	0.31
Maximum	11.3	1650	1610	435	535	320	3.66	1.05	8.71	448	44.6	2270	3.82
PCV (or WHO^d,e^)	6.5 and 9.5	2500	200	10	1000	700^d^	12^e^	2^e^	5	250	50	2000	1.5
>PCV (*n*)	231^c^	0	34	27	0	0	0	0	1	1	0	1	3
>PCV (%)	47	0	7	5	0	0	0	0	0	0	0	0	1

	Fe	Mn	Mo	Na	Ni	NO_2_	NO_3_	Pb	Sb	Se	SO_4_	U
Method	ICP-MS	ICP-MS	ICP-MS	ICP-MS	ICP-MS	IC	IC	ICP-MS	ICP-MS	ICP-MS	IC	ICP-MS
Units	µg/L	µg/L	µg/L	mg/L	µg/L	mg/L	mg/L	µg/L	µg/L	µg/L	mg/L	µg/L
Detection limit	1	0.2	0.03	0.2	0.1	0.01	0.02	0.02	0.005	0.1	0.05	0.002
Minimum	<1	<0.2	<0.03	1.2	<0.1	<0.01	<0.02	<0.02	<0.005	<0.1	<0.05	<0.002
5th percentile	<1	<0.2	<0.03	8.84	0.1	<0.01	0.05	0.03	0.008	<0.1	4.61	0.003
25th percentile	1.04	0.86	<0.03	13.3	0.31	<0.01	7.40	0.15	0.020	0.11	9.39	0.017
50th percentile	3.27	5.04	0.05	18.1	0.83	<0.01	17.6	0.33	0.042	0.31	14.6	0.075
75th percentile	11.8	17.8	0.12	25.5	2.80	<0.01	33.1	0.74	0.107	0.59	23.1	0.491
95th percentile	113	270	0.63	46.3	17.6	0.03	64.4	2.90	0.507	1.41	44.4	2.02
Maximum	6300	2030	11.6	164	115	0.34	140	44.5	36.8	8.54	141	11.9
PCV (or WHO^d,e^)	200	50	70^e^	200	20	0.5	50	10	5	10	250	30^d^
>PCV (*n*)	15	59	0	0	15	0	53	5	6	0	0	0
>PCV (%)	3	12	0	0	3	0	11	1	1	0	0	0

^a^ *n* = 494

^b^ *n* = 495. All other analytes, *n* = 497

^c^ *n* within pH PCV range

^d^WHO guideline value (including provisional values)

^e^WHO non-formal health-based value

### Exceedances of parameter threshold values

The parameters which most frequently fall outside the PCV (or other threshold) are: pH (47 %); Mn (12 %); NO_3_ (11 %); Al (7 %); As (5 %); Fe (3 %); and, Ni (3 %). These are summarised in Table 2 and Fig. 3. Other parameters either have ≤6 (≤1 %) failures, or for 12 parameters there were no failures measured in this study.

### Exceedances in individual water samples

**Fig. 4:**
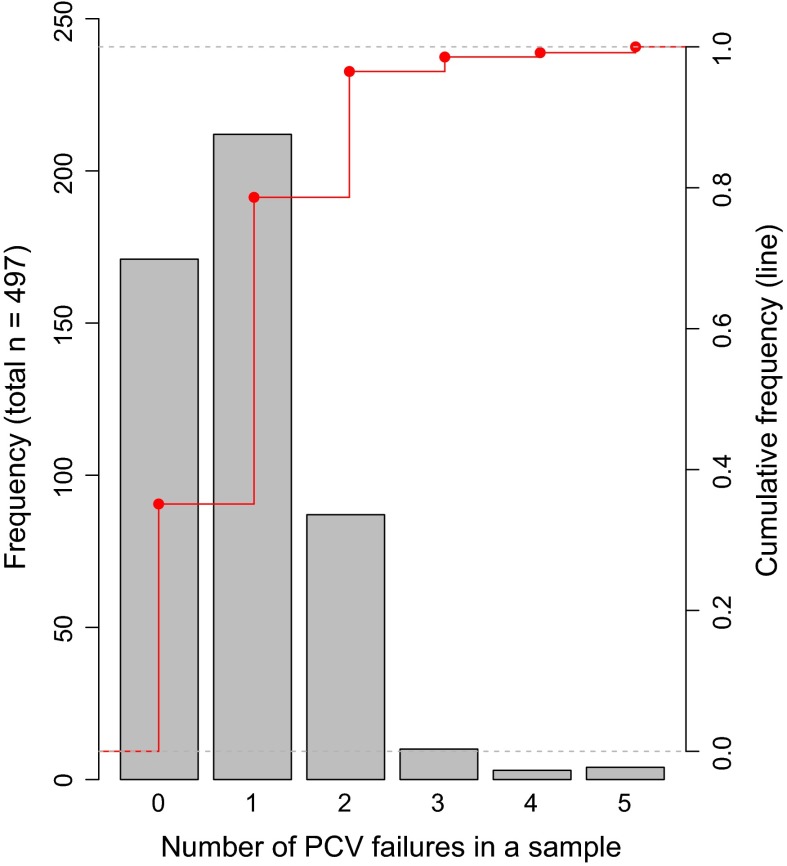
Frequency of the number of PCV failures per sample as total count and cumulative frequency

The overall count of household drinking water samples shows that 35 % (*n* = 171) do not fail any of the standards for the 25 parameters reported in this study. The greatest frequency occurrence, at 44 % (*n* = 212), was for a single exceedance against any one of the PCVs (Fig. 4). The remaining 21 % (*n* = 104) have two or more PCV failures, with four samples having five failures.

**Fig. 5:**
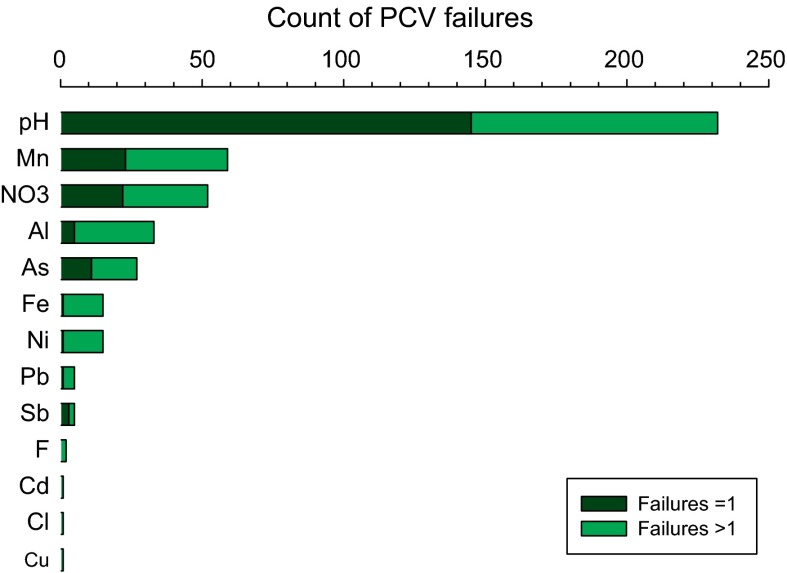
Sample counts where PCV failures for each parameter occur as a single failure for a sample, or as one of two or more failures for a sample

Of the 32 different permutations of multiple PCV failure arising in these data, only three unique combinations of dual exceedances occurred more than 10 times. These were pH and: NO_3_ (*n* = 25), Al (*n* = 20) or Mn (*n* = 15). There were 12 concurrent failures of Fe and/or Mn, in various combinations with other elements. The most frequent multiple failure for As was in conjunction with pH (*n* = 10) and for Ni was in conjunction with Mn (*n* = 9), both in various permutations with other parameters. pH had the numerically (*n* = 145) and proportionally (63 %) largest incidence of single PCV failure. The proportions of PCV failures which occur singly, or in a combination, are shown in Fig. 5. This shows where a parameter was the only case of a PCV failure for a given sample; this was highest numerically and proportionally for pH (63 %), NO_3_ (42 %), As (41 %) and Mn (39 %) so other than for pH, the majority of PCV failures were in combination with one or more other PCV parameter.

### Unfiltered and filtered comparison

At a small number of taps, with no treatment system in place, samples were collected as unfiltered and filtered pairs. These were collected at the same time, and differences between concentrations in them may be indicative of particulate transport. There was no detectable difference between the paired samples, in the majority of the 23 measured parameters (Suppl. Figure 1). The exceptions to this were Fe and Pb. The majority of the Fe samples were indistinguishable in concentration, other than five of the 29 sample pairs where unfiltered concentrations were greater. Lead concentrations were indistinguishable in 20 of the sample pairs, whilst the remaining nine sample pairs showed variable relationships between filtered and unfiltered concentrations (Suppl. Figure 1).

## Discussion

### Nitrate concentrations and nitrate removal treatment systems

The 11 % exceedance of the NO_3_ PCV in this dataset was the highest failure rate of a health-based ‘chemical parameter’ (DEFRA 2009; WHO 2011a). However, the proportions from some other UK studies are higher: compiled private water supply summary NO_3_ data for England found 17 % (200/1160) above the PCV (Drinking Water Inspectorate 2015), whilst it was 21 % from one English region (Harrison et al. 2000), and 15 % in one Scottish county (Reid et al. 2003). Whilst inputs of NO_3_ from agricultural or septic tank sources may be expected, denitrification process will also remove NO_3_ from groundwater, causing the bimodal data distribution that was observed in English and Welsh groundwater (Shand et al. 2007) and is also seen in these data (c.f. Fig. 3). This is also evidenced by the mutually exclusive occurrence of high Fe or Mn and high NO_3_ concentrations in these data, with high Fe concentrations being indicative of reducing groundwater.

Two samples had NO_3_ concentrations of ~140 mg/L, with the remainder of the samples above the PCV falling in the range 50–100 mg/L (Fig. 3). The concurrent failure of NO_3_ with low (<6.5) pH drinking water is not considered causative, but indicative of the naturally low-pH environment of much of the area where oxidising, nitrate-containing, groundwaters are extracted. Where drinking water concentrations exceed 50 mg/L, this is likely to result in drinking water being the largest dietary source of NO_3_ (Chilvers et al. 1984). Where concentrations exceed 50 mg/L, the use of alternative water for infant formula is recommended, to prevent methaemoglobinaemia (“blue baby syndrome”) (Drinking Water Inspectorate 2011).

Of the 53 drinking water samples with >50 mg/L, participants at 12 properties volunteered the information that they knew of high NO_3_ in their drinking water. Of those 12, five had NO_3_ removal systems (point-of-use) installed and a measured NO_3_ concentration of 54–78 mg/L. A further subset of three of these had paired groundwater and drinking water samples, which were 60 and 62, 56 and 58, 78 and 79 mg/L, respectively, showing no difference between the ‘treated’ drinking water and the source groundwater, as observed elsewhere (Reid et al. 2003). Two further sampled properties had NO_3_ point-of-use devices installed and had much lower NO_3_ concentrations of 1 and 12 mg/L, although at these properties no untreated groundwater sample was collected, so the effect of treatment cannot be confirmed. The point-of-use nitrate removal systems generally recommended for private water supply systems are ion-exchange and require maintenance every 5 days to ensure their efficacy (Scottish Executive 2006). It is surmised that some householders are not undertaking maintenance as specified for their system and that this is rendering the units ineffective.

### Arsenic

Of the substances measured in this survey which are categorised as ‘chemical parameters’ in national and EU legislation, the second greatest proportion of PCV failures, at 5 % (*n* = 27), was found for As. This proportion is very similar to the 6 % (*n* = 14/249 for 2010–2014) reported in a national compilation of available data on single domestic dwellings (Drinking Water Inspectorate 2015). Whilst the high concentrations of As in other sample media (soils, surface water, stream sediments, mine waters) in Cornwall have long been documented and are widely recognised (Abrahams and Thornton 1994; Ander et al. 2013; Aston et al. 1975), there were no public domain pre-existing data on private drinking water supply As concentration in Cornwall. All data (*n* = 76) for the baseline survey of granite aquifers in south-west England were <6 µg/L for As (Smedley and Allen 2004), and the overall median for groundwater in England and Wales was <1 µg/L (Shand et al. 2007). Thus, this study provides new information on concentrations >100 µg/L (*n* = 4) and >10 µg/L (*n* = 27) in private water supplies in this area and finds 29 % (*n* = 145) of samples >1 µg/L. A US Geological Survey compilation study (*n* = 1774 for As) of data for principal aquifers found a remarkably similar proportion of samples >10 µg/L, 6.8 %, to this study (DeSimone et al. 2009), although in some areas of north-east USA this rises to 13–17 % in whole-state studies (Ayotte et al. 2003; Peters et al. 2006). Typical concentrations, and proportions of samples >100 µg/L, in this area are fortunately lower than those in areas of the world with serious health effects from high drinking water As (e.g., Smedley and Kinniburgh 2002; Murcott 2012; Vaughan and Polya 2013). However, there is increasing evidence of association between prolonged exposure, via drinking water, to As concentrations <100 µg/L and specific disease outcomes, e.g., basal cell carcinoma (Leonardi et al. 2012) and squamous cell carcinoma (Gilbert-Diamond et al. 2013). This is also recognised in the provisional nature of the WHO As GV of 10 µg/L, which is based on limitations of available treatment and measurement of aqueous As when the most recent guideline values were produced (WHO 2011a).

There is no evidence in a tested subset of these data of particulate As transport (Suppl. Figure 1), which was found by Copeland et al. (2007) from pipe corrosion in public supplies. Peters et al. (1999) found an 11 % difference between filtered and unfiltered As in private well supplies, but a time-lag of 1–12 months between collection of those samples meant that although they considered that some As was likely to be as particulate phases, they could not rule out groundwater concentration fluctuations.

**Fig. 6:**
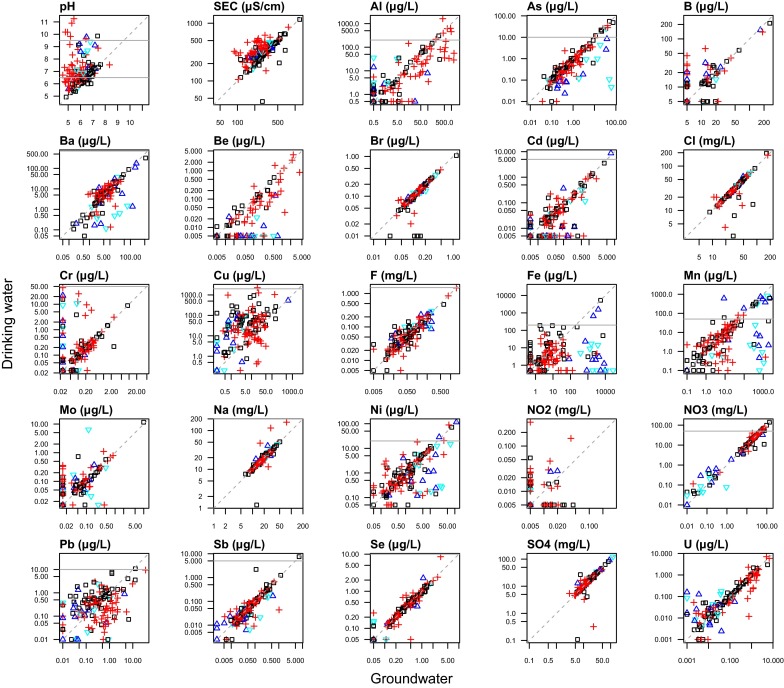
Comparison of paired groundwater and drinking water sample data with ‘pH adjustment’ and ‘Fe/Mn removal’ treatment systems reported by householder. *Black squares*—neither treatment; *red crosses*—pH adjustment; *blue triangles*—FeMn removal; *pale blue inverted triangle*—both treatments; *grey horizontal line*—PCV or GV (this is not shown where axis maximum value is below this value)

**Fig. 7:**
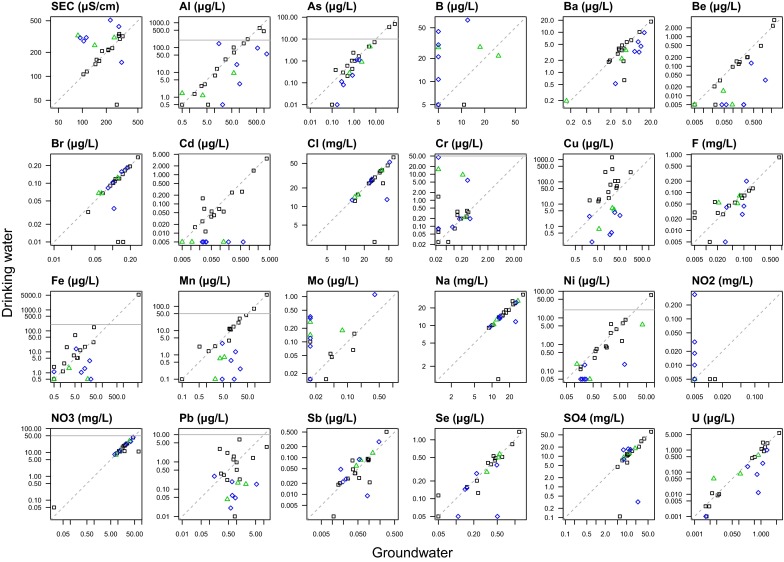
Comparison of groundwater and drinking water data where pH adjustment of acid groundwaters is not used (*black squares*), or alters drinking water pH to 8.5–9.5 (*blue diamonds*) or to pH > 9.5 (*green triangles*). *Dashed grey line* shows the line of equivalence. *Solid horizontal grey line* shows PCV or WHO values, where these are below the axis maximum value. Summary statistics for each dataset are provided in Suppl. Table 1

Treatment options to decrease As concentrations are available for domestic private water supplies (Scottish Executive 2006). In this study area, two properties reported treatment systems installed due to high As in the source groundwater; the groundwater and drinking water concentrations for these was 14 and 49 µg/L, 1.0 and 0.1 µg/L, respectively, demonstrating the success of both the systems and their effective operation. These samples both had concurrent high Fe (11,000 and 1500 µg/L) in groundwater, and a wider adventitious decrease in As concentrations of paired samples where Fe and/or Mn removal is used can be seen (Fig. 6), as is the case for Ni. The precipitated Fe and Mn minerals are likely to promote As co-precipitation (Smedley and Kinniburgh 2002). Additionally, samples where pH treatment is used (Fig. 7) show a small, but significant, decrease (one-sided, paired *t* test, *p* = 0.003) unlike the untreated, acid, waters (paired *t* test, *p* = 0.43). The difference is, however, small and appears to result from a small number of data points at lower starting As concentrations (<1 µg/L).

In the 27 samples with As >10 µg/L, there were 11 (41 %) which only had As above the PCV, and a further six where both pH and As were outside the PCV range. Arsenic does not impart discolouration nor flavour to water when it occurs at high concentrations (Zheng and Ayotte 2015), making detection without testing impossible. There is a considerable literature developing on the inhibitions that exist to installing and maintaining treatment systems, even in areas with a greater proportion of high As concentrations than found in this study (e.g., Zheng and Ayotte 2015).

### pH and treatment of low-pH groundwaters

The pH data in this study have the highest PCV failure rate of any parameter studied, at 47 %, despite 39 % of samples being reported to have specific pH treatment systems in place. Of these 42 % (*n* = 208) were for low (<6.5) pH, whilst the remainder were for high pH values (>9.5). The English summary data (Drinking Water Inspectorate 2015) report a lower failure rate of 15 % (*n* = 281/1859). Whilst there is some evidence that particularly low pH (<4) values may be of direct concern for health through effects on external organs (eye, skin) (WHO 2003b), the lowest value encountered in this survey was higher than that, at 4.8.

### Metals from household plumbing systems

Copper concentrations increase between source groundwater and drinking water sample points where pH stays <6.0, whilst they typically decrease where pH is raised to >8.5 (Fig. 7). Copper is a common plumbing pipe material in this area and decreased corrosion when pH is increased is consistent with treatment recommendations (e.g., Scottish Executive 2006). Copper corrosion may cause premature leaks in plumbing systems (including central heating), and oxide or carbonate precipitates may stain bathroom and kitchen fittings, laundry (Scottish Executive 2006) and stain dyed-hair (as reported by several householders in this study). The inconvenience of this may contribute to the measurement and higher incidence (5 %, *n* = 23/516) of Cu PCV exceedances in the summary English private drinking water data compiled by the Drinking Water Inspectorate (2015).

Corrosion of other metals from pipes and fittings is widely recognised (Gonzalez et al. 2013; Tam and Elefsiniotis 2009), although most studies focus on public distribution systems, with much longer pipe lengths. Interestingly, no systematic increase in Cd, Cr or Ni was seen in the pH < 6.0 drinking waters, although this has been seen in other studies where metal fixtures and fittings are used (Gonzalez et al. 2013). Particulate transport of metals can arise from naturally occurring particulates or detachment of mineral flakes from precipitates within pipes, particularly under high flow rates (Hulsman 1990; Clark et al. 2014; Dinelli et al. 2012). Where drinking water (unfiltered) concentrations exceed those of the source groundwater (filtered) sample, such as for Fe and Pb (Fig. 7) one plausible explanation is that drinking water data include metals from particulates. This is also supported by the data from paired filtered and unfiltered samples (Suppl. Figure 1). There is no evidence here of liberation of elements such as As or U from treatment or pipe network as has been found in municipal supply systems (Copeland et al. 2007; Lytle et al. 2014).

### Wider effects of pH adjustment

Where treatment has increased pH from acidic (<6.5) to alkaline (>8.5), a wider impact is seen across a suite of parameters other than Cu, with lower drinking water concentrations of Al, As, Ba, Be, Cd, Fe, Mn, Ni and Pb (Fig. 7). The effects on As, Fe and Mn are discussed elsewhere.

The Al 200 µg/L PCV is set for aesthetic reasons (precipitation of Al salts) and is largely derived from expectations of Al-containing coagulants being used in water treatment works (WHO 2011a): a consideration irrelevant to the properties sampled in this study. WHO do not set a health-based guideline because it would be 900 µg/L, i.e. higher than the aesthetic control recommendation (WHO 2011a, p. 311). One drinking water sample exceeded 900 µg/L in this survey, and although there is a compositional gap in the data at this concentration (Fig. 3), there are seven samples >500 µg/L. Natural conditions are responsible for the 7 % of samples above the 200 µg/L PCV in this dataset, as illustrated by the indistinguishable paired Al data in samples with unaltered, acid, pH (Fig. 7, paired *t* test, *p* = 0.23). This is why the proportion of Al samples with concurrent PCV failures >1 is 63 % (Fig. 5): they are coincident with pH <6.5. However, Fig. 7 shows that where pH has been increased to >8.5, concentrations are significantly lower (one-sided paired *t* test, *p* < 0.05), which is consistent with decreased Al solubility as acidity decreases (Tipping 2005).

There are substantial decreases in Be, Cd and Ni concentrations when pH is treated to a value of >8.5, with the majority of data below the detection limit (Fig. 7). This is particularly interesting for Cd and Ni where concentrations were found at or above the PCV.

Notably, the alteration to pH > 8.5 is seen to have the effect of increasing the concentrations of a small number of parameters. The increase in conductivity (Fig. 7) is a natural consequence of the dissolution of a mineral which is being used to buffer the pH to a higher value. Given the concomitant increased Ca, Mg and HCO_3_ concentrations (not presented), these are indicative of mixed Ca, Mg carbonate or hydroxide minerals as the ameliorant. In addition to conductivity, there are also intermittent, or systematically, increased B, Cr, Mo and NO_2_ concentrations (Fig. 7), although the data for the latter are inconclusive since all are very close to the detection limit. The geochemistry of B, Cr and Mo is such that they are all preferentially mobile as oxyanion species at alkaline pH values (Smedley et al. 2002; Smedley et al. 2014a). The proportional increase for B would seem to be greatest at pH > 9.5, although sample numbers become small at this stage of comparison (Fig. 7). Carbonates, such as chalk, provide a significant geological sink for B (Vengosh et al. 1991), so this may indicate a relationship with the amount of mineral dissolution required to buffer the pH. Whilst there are up to tenfold increases in B concentrations, all are still substantially below the PCV of 1000 µg/L. The relative increase in Mo is similar and likewise does not increase concentrations to values which are close to 70 µg/L (Fig. 7), which is consistent with the findings of Smedley et al. (2014a), nor is it likely to be associated with particulate Mo phases (Suppl. Figure 1). The concentrations of drinking water Cr are more variable, with five of the nine samples having concentrations unaltered by pH correction. The remaining four samples have concentrations 5.9–45 µg/L in drinking water, approaching the PCV of 50 µg/L (Fig. 7). Whilst increased mobility for Mo and Cr at increased pH values is expected, the underlying source of increased concentrations is not currently resolved.

### pH over-correction

There is limited historical data suggesting that higher pH values, perhaps pH > 10, could affect both eyes and/or skin and potentially have gastrointestinal effects in some individuals (reference unavailable, but cited in WHO 2003b), but WHO have not set a health-based GV. pH adjustment systems led to 5 % of drinking water samples having pH > 9.5, and of these there were seven households (1 %) using drinking water with a pH ≥ 10. These high pH values arise from over-correction of naturally low-pH groundwater sources, and it is not known how long the high pH conditions typically persist at a property. Whilst there are presumably cost implications of this over-correction, it is not clear to what extent the higher pH values are of interest from a public health perspective. There may also be implications for the efficacy of other treatment systems, e.g., where chlorine is used for disinfection, pH should be <8 (WHO 2003b).

### Iron and manganese and their treatment

Groundwater Mn concentrations have been shown to frequently exceed 50 µg/L (Homoncik et al. 2010; Shand et al. 2007), and this variability in the British context is as seen on the global scale (e.g., Frisbie et al. 2012). This study translates those findings into household drinking water use and shows that it is reasonable to assume that high groundwater Mn indicates the possibility of high drinking water Mn where household private water supplies are used—albeit moderated by treatment system choices.

Whilst a low proportion (3 %) of sample data were above the WHO Mn health-based GV of 400 µg/L (WHO 2011a), a greater proportion of households were using higher Mn concentration drinking water than in studies cited in the background to the WHO drinking water guidelines (WHO 2011b). Furthermore, the findings of this study do not fully support assumptions that 400 µg/L is unlikely to be exceeded, and also that neither 50 µg/L (c.f. WHO 2011b, p. 15) nor 100 µg/L (WHO 2011a, p. 226) will necessarily act as upper limits to acceptability (water colour, scaling), with 9 % of samples ≥100 µg/L. Whilst the essential nutritional role and requirement of Mn is recognised, there has been some debate as to whether the WHO health-based GV of 400 µg/L is sufficiently protective for vulnerable receptors, particularly infants (Ljung and Vahter 2007; Frisbie et al. 2012). Here we find that 57 % (*n* = 17/30) of paired samples had starting concentrations that became <50 µg/L in drinking water following point-of-entry treatment (Fig. 6), confirming that domestic treatment can be effective, as for other parameters (Scottish Executive 2006).

However, some 18 households reported Fe and/or Mn treatment systems installed, but all exceeded the Mn PCV and two the Fe PCV, probably indicating poor system maintenance. Compiled private drinking water data for England found 18 % (*n* = 236/1351) above the Mn PCV (Drinking Water Inspectorate 2015), which is somewhat higher than the 12 % in this study. The PCVs for both Mn and Fe are based on water visual properties (discolouration) and protection of pipework from scaling (WHO 2011a).

Iron also has an optimal intake range for nutrition, but at high concentrations can be harmful (WHO 2011a). A noticeable taste and staining of laundry and plumbing at >300 µg/L are considered to dissuade people from using drinking water that would present potential health concerns (WHO 2011a). Highly variable natural groundwater concentrations are widely found (Shand et al. 2007), but there were five drinking waters being used which had >1000 µg/L Fe. Thus assumptions of protection conferred by avoidance of drinking water sources with Fe > 300 µg/L due to an adverse taste and discolouration (WHO 2003a) may not be supported.

### Fluoride

Fluoride PCV (1.5 mg/L) exceedances in the drinking waters were at a very similar percentage to those found in the national compilation data, which was 2 % (Drinking Water Inspectorate 2015). Higher concentrations of F in this study were generally associated with low Ca waters, a condition which promotes F solubility (Edmunds and Smedley 2013), although it should be reiterated that only three samples were >1.5 mg/L, with a maximum concentration of 3.8 mg/L. Higher concentrations of F in this study were not affected by treatment systems employed in this area (Fig. 6), despite pH adjustment systems being likely to be based on calcium carbonate buffering of pH, which would be expected to reduce F concentrations (Edmunds and Smedley 2013). Drinking water F is beneficial to tooth enamel at lower concentrations than the PCV, with 0.5 mg/L considered to be the lower beneficial concentration, although at concentrations much above the PCV detrimental effects, such as dental fluorosis, are recognised (Fawell et al. 2006).

### Other measured parameters

The remaining parameters were found to have very low rates of PCV exceedance (Cl, Na, NO_2_, Sb, Se, SO_4_), or to be universally below the WHO values. Neither Sb nor Se have concentrations systematically decreased by any of the treatment systems widely reported in this study (Fig. 6). Whilst the exceedance rate was low for Sb (*n* = 6; 1 %) and there were no samples >10 µg/L for Se, this should continue to be monitored in any surveillance data from other areas. Elements such as Sb may also have synergistic relationships with health outcomes in high As areas (Frisbie et al. 2009).

None of the elements compared here to WHO values (Ba, Be, Br, Mo and U), had any exceedances in this study, although all had maximum values within twofold or threefold the WHO value (Table 2). There is no action that is required as a result of these findings, but these data provide valuable background information on parameters which could in future be incorporated into EU regulations.

Uranium concentrations were lower than those found in English and Welsh groundwater, which had a median of 0.27 µg/L (*n* = 869) (Shand et al. 2007), although local granite groundwater were previously reported to have a median U concentration of 0.55 µg/L (Smedley and Allen 2004). The maximum value, 12 µg/L, was below the provisional health-based GV (30 µg/L) (WHO 2011a), but closer to the previous GV of 15 µg/L (WHO 2004). These concentrations are lower than those found in other private drinking water supplies, e.g., 17 % of wells in a Swedish study were >15 µg/L (Norrström and Löv 2014). At higher U concentrations observed in this study (>0.1 µg/L), drinking water concentrations were unaffected by treatment systems (Fig. 6), although installations were not specific for removal of U. The recommended U removal methods for private water supplies are ion-exchange and membrane filtration, although the former can be compromised by the dominance of uncharged U species at circum-neutral pH (Norrström and Löv 2014).

### Caveats

The study design employed was random sampling, but this may have been modified by the voluntary participation. Further work will need to assess whether there is excess clustering in some areas before being able to draw spatial statistical interferences about concentrations at unsampled locations.

This study was a one-off survey conducted at two separate intervals: it is assumed that variation within the dataset during the survey is substantially greater than variation in time, which is supported by the majority of study findings that have investigated this aspect (Slotnick et al. 2006; Ayotte et al. 2015), although further monitoring would be required for confirmation.

## Conclusions

These new data for 497 tap water samples in a mineralised area (Cornwall, UK) reveal that considerable variation exists in drinking water quality and the treatment system choices householders make about their private water supply. The random study design decreases the opportunity for bias in outcomes, providing a sound basis for future decision making in relation to the proportional exceedances of water quality standards which have been found. The proportion of samples with one or more failures (65 %) is greater than those with no exceedances for the 25 chemical parameters reported in this study, and multiple concurrent exceedances were found in 21 % of samples. Householders were given public health advice where there were exceedances and support from the regulator (the local authority) was provided regarding remedial measures.

For the parameters with health-based drinking water standards, the most frequently exceeded were NO_3_ (11 %) and As (5 %). Despite the lower percentage exceedance of As, it is probably the most important because the highest concentration found was 440 µg/L which is substantially greater than the 10 µg/L standard, and health risks are thought to increase with higher concentrations and longer duration of exposure. Nitrate concentrations are considered to be of greatest concern to bottle-fed infants once the water concentration exceeds 50 µg/L due to the risk of methaemoglobinaemia, particularly where the water may not be microbiologically safe. Treatment systems installed intentionally to remove either of these constituents were rarely reported, and in the case of NO_3_ were demonstrably unsuccessful in three of five instances. No other treatment systems decreased NO_3_. However, unsurprisingly, adventitious decreases in As concentrations were found where treatment to decrease Fe and/or Mn concentrations were being used.

The greatest rate of exceedance was that of pH (47 %), which was found to be both naturally low (42 %) and treated to excessively high values (5 %) in this area. Where pH values were low, increased Cu concentrations in drinking water indicated pipework corrosion, whilst successful pH treatment is associated with concomitant decreased Al, Cd, Cu, Pb and Ni in drinking water. Thus, the 31 % of samples for which pH treatment was reported as installed, but still had drinking water values <6.5 illustrate the scale of missed opportunities to have multiple benefits on drinking water quality through suboptimal system maintenance.

Naturally elevated Fe and Mn were found in drinking water sources, and in some cases exceeded the drinking water standard where untreated. Unpleasant odour, unpalatability and staining are assumed to confer protection from high Mn or Fe drinking water. However, the second highest exceedance in this study was Mn (12 %), and a small number were being used with Mn concentrations exceeding the WHO health-based value of 400 µg/L. This study provides evidence that the common assumptions about limitations to acceptability of drinking water may not be universally true.

Understanding naturally occurring variations in inorganic constituents in private drinking water supplies is a vital part of ensuring protection to populations where these form the sole source of drinking and cooking water to households, as this forms a direct route of exposure. Further work will be undertaken in this region to support the local regulator in public information dissemination and opportunities for informed decision-making by householders on options for improving their water quality. This study reinforces the importance of householder water quality testing and understanding of system maintenance requirements. From this work, population exposure is being studied (Crabbe et al. in press; Middleton et al. subm.), and a risk assessment based on underlying geology is underway, to help quantify the public health burden of chronic exposures to chemicals in private water supplies in Cornwall. This pilot project methodology has much wider potential to define and prioritise areas for further investigation and demonstrates the potential for poor drinking water quality even in a region where public (mains) water supplies are widely available, and the use of private water supplies often reflects choice rather than necessity.

## Electronic supplementary material

Below is the link to the electronic supplementary material.

Suppl. Figure 1 Comparison of drinking water samples collected as both 0.45 µm filtered and unfiltered at the same sample point (n = 29). (PDF 55 kb)

Supplementary material 2 (DOCX 16 kb)
