# Supplementary figures and images for: Variability in the chemistry of private drinking water supplies and the impact of domestic treatment systems on water quality
Source: Environ Geochem Health. 2016 Jan 25;38(6):1313–32. doi: 10.1007/s10653-016-9798-0 (PMC5095163; doi:10.1007/s10653-016-9798-0)

Unfiltered sample

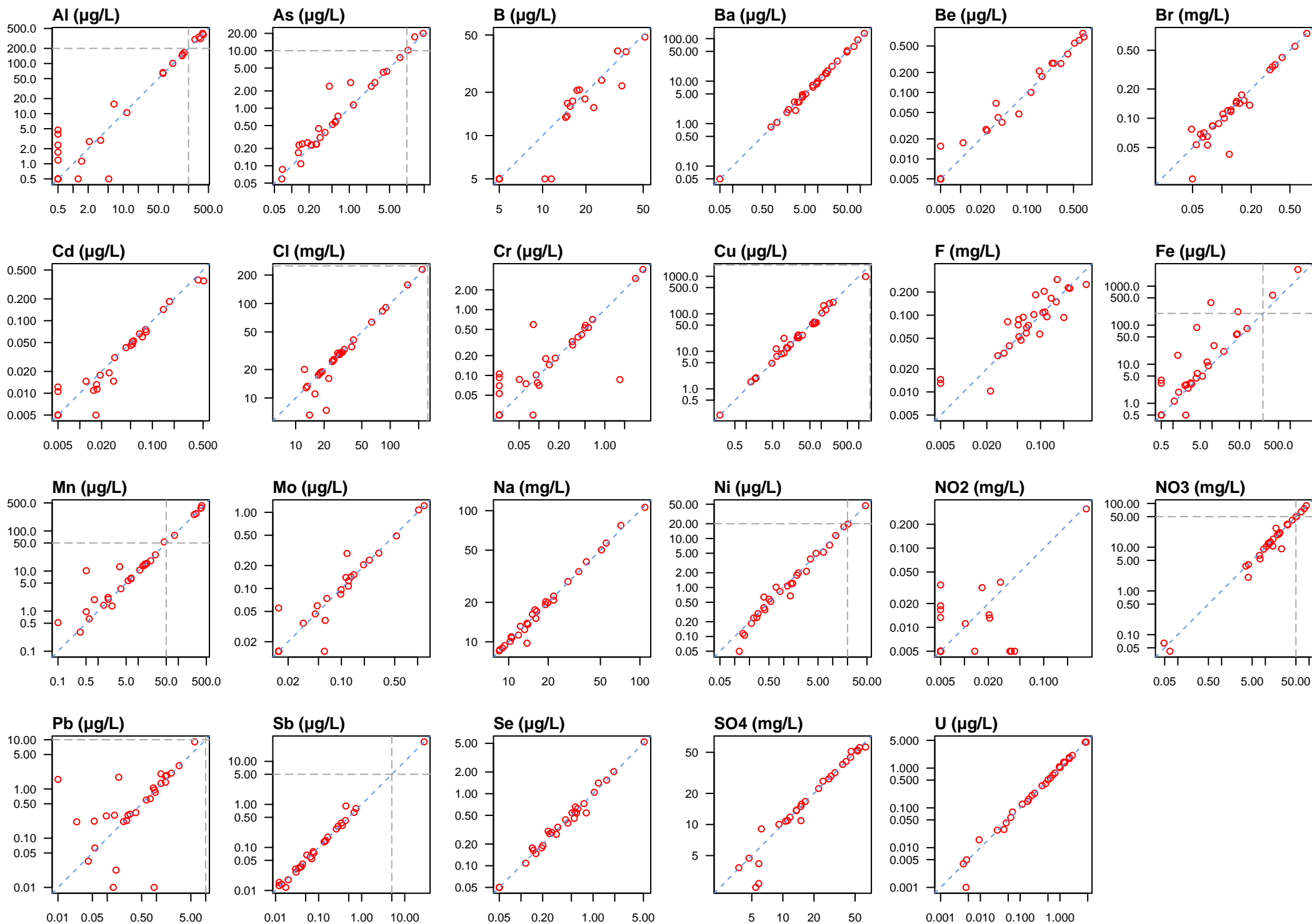

0.45  $\mu\text{m}$  filtered sample

Supplement: Supplementary file 1 — Comparison of drinking water samples collected as both 0.45 µm filtered and unfiltered at the same sample point (n = 29). (PDF 55 kb) [file 10653_2016_9798_MOESM1_ESM.pdf]
