# Supplementary material for: Variability in the chemistry of private drinking water supplies and the impact of domestic treatment systems on water quality
Source: Environ Geochem Health. 2016 Jan 25;38(6):1313–32. doi: 10.1007/s10653-016-9798-0 (PMC5095163; doi:10.1007/s10653-016-9798-0)
Supplement: Supplementary file 2 — Supplementary material 2 (DOCX 16 kb) [file 10653_2016_9798_MOESM2_ESM.docx]

Supplementary Table 1: Summary statistics for data presented in Figure 7, showing comparison between untreated and pH adjusted sample pairs as described in Figure 7 caption.

| Group | High drinking water pH | | | | | | Low drinking water pH | | | | | |
| --- | --- | --- | --- | --- | --- | --- | --- | --- | --- | --- | --- | --- |
| Water Type | Unfiltered (drinking water) | | | Filtered (groundwater) | | | Unfiltered (drinking water) | | | Filtered (groundwater) | | |
| *Percentile* | *25^th^* | *50^th^* | *75^th^* | *25^th^* | *50^th^* | *75^th^* | *25^th^* | *50^th^* | *75^th^* | *25^th^* | *50^th^* | *75^th^* |
| pH | 9.17 | 9.73 | 10.0 | 5.14 | 5.25 | 5.81 | 5.43 | 5.48 | 5.54 | 5.10 | 5.39 | 5.45 |
| SEC (µS/cm) | 278 | 306 | 317 | 104 | 128 | 239 | 142 | 162 | 243 | 151 | 212 | 275 |
| Al (µg/L) | 1.4 | 6.4 | 58.1 | 15.2 | 40.9 | 92.2 | 3.3 | 101 | 224 | 5.56 | 61.1 | 280 |
| As (µg/L) | 0.11 | 0.59 | 1.12 | 0.33 | 1.09 | 1.99 | 0.30 | 1.01 | 10.6 | 0.32 | 0.94 | 11.0 |
| B (µg/L) | 21.0 | 24.6 | 29.0 | 5 | 5 | 14.7 | 5 | 5 | 5 | 5 | 5 | 5 |
| Ba (µg/L) | 2.19 | 3.27 | 3.44 | 3.74 | 6.29 | 8.73 | 1.90 | 3.67 | 6.82 | 3.20 | 4.15 | 6.33 |
| Be (µg/L) | <0.005 | <0.005 | 0.024 | 0.047 | 0.077 | 0.254 | 0.096 | 0.139 | 0.277 | 0.097 | 0.155 | 0.454 |
| Br (mg/L) | 0.068 | 0.079 | 0.104 | 0.079 | 0.100 | 0.118 | 0.032 | 0.101 | 0.148 | 0.102 | 0.124 | 0.153 |
| Cd (µg/L) | <0.005 | <0.005 | <0.005 | 0.024 | 0.030 | 0.082 | 0.037 | 0.055 | 0.112 | 0.032 | 0.050 | 0.163 |
| Cl (mg/L) | 14.2 | 17.4 | 23.5 | 15.5 | 24.7 | 35.4 | 15.6 | 26.5 | 36.3 | 26.5 | 27.4 | 36.4 |
| Cr (µg/L) | 0.19 | 0.21 | 12.47 | 0.03 | 0.13 | 0.24 | 0.06 | 0.24 | 0.29 | 0.03 | 0.14 | 0.22 |
| Cu (µg/L) | 0.5 | 2.9 | 4.9 | 5.8 | 20.4 | 28.5 | 34.6 | 53.7 | 83.1 | 11.1 | 15.9 | 31.4 |
| F (mg/L) | 0.041 | 0.053 | 0.084 | 0.034 | 0.068 | 0.094 | 0.055 | 0.057 | 0.084 | 0.028 | 0.058 | 0.093 |
| Fe (µg/L) | 0.50 | 1.37 | 1.64 | 2.60 | 7.82 | 12.29 | 2.82 | 5.16 | 24.55 | 1.53 | 5.10 | 7.30 |
| Mn (µg/L) | 0.10 | 0.65 | 1.07 | 4.48 | 5.01 | 7.92 | 3.88 | 12.04 | 52.52 | 8.55 | 10.19 | 55.55 |
| Mo (µg/L) | 0.120 | 0.226 | 0.347 | 0.015 | 0.015 | 0.046 | 0.015 | 0.015 | 0.060 | 0.015 | 0.015 | 0.022 |
| Na (mg/L) | 11.6 | 12.9 | 13.4 | 12.0 | 13.5 | 19.5 | 10.1 | 16.5 | 19.8 | 12.8 | 15.9 | 20.1 |
| Ni (µg/L) | 0.05 | 0.11 | 0.18 | 0.20 | 0.24 | 3.99 | 0.56 | 0.68 | 2.58 | 0.61 | 0.68 | 6.15 |
| NO_2_ (mg/L) | <0.005 | <0.005 | 0.012 | <0.005 | <0.005 | <0.005 | <0.005 | <0.005 | <0.005 | <0.005 | <0.005 | <0.005 |
| NO_3_ (mg/L) | 10.1 | 11.7 | 25.6 | 10.9 | 12.0 | 28.2 | 11.0 | 11.3 | 20.6 | 12.5 | 18.9 | 28.9 |
| Pb (µg/L) | 0.05 | 0.15 | 0.18 | 0.61 | 0.80 | 1.65 | 0.37 | 0.96 | 1.59 | 0.79 | 0.86 | 1.75 |
| Sb (µg/L) | 0.03 | 0.07 | 0.11 | 0.02 | 0.06 | 0.11 | 0.03 | 0.04 | 0.06 | 0.04 | 0.04 | 0.06 |
| Se (µg/L) | 0.14 | 0.28 | 0.39 | 0.15 | 0.41 | 0.51 | 0.13 | 0.41 | 0.52 | 0.23 | 0.35 | 0.49 |
| SO4 (mg/L) | 8.0 | 10.8 | 16.3 | 8.1 | 12.1 | 17.5 | 6.2 | 11.9 | 13.2 | 7.2 | 10.4 | 13.1 |
| U (µg/L) | 0.051 | 0.132 | 0.422 | 0.116 | 0.458 | 0.980 | 0.003 | 0.884 | 1.38 | 0.005 | 1.06 | 1.26 |
